# The efficacy and safety of acupuncture and moxibustion combined with western medicine for obsessive-compulsive disorder: A protocol for systematic review and meta-analysis

**DOI:** 10.1097/MD.0000000000021395

**Published:** 2020-08-28

**Authors:** Chunying Tian, Yihua Fan, Jingyu Xu, Yang Huang, Wen Wang, Shenjun Wang, Ruiwen Song, Xinju Li

**Affiliations:** a Tianjin University of Traditional Chinese Medicine; b First Teaching Hospital of Tianjin University of Traditional Chinese Medicine; c National Clinical Research Center for Chinese Medicine Acupuncture and Moxibustion, Tianjin, P. R. China.

**Keywords:** acupuncture, moxibustion, obsessive-compulsive disorder, protocol, systematic review

## Abstract

**Background:**

Obsessive-compulsive disorder is common, chronic mental disorder, which is characterized by recurrent, unwanted, or intrusive thoughts and repetitive behaviors or mental action. Acupuncture and moxibustion, as a popular form of complementary and alternative therapy, have the advantages of low side effects, high safety, and low cost. The research showed that acupuncture and moxibustion have a good clinical efficacy on obsessive-compulsive disorder. However, there is no literature to systematically evaluate the efficacy and safety of acupuncture and moxibustion in treating obsessive-compulsive disorder. Thus, this study is aimed to evaluate the efficacy and safety of acupuncture and moxibustion for obsessive-compulsive disorder patients, providing reliable evidence for clinical application.

**Methods:**

Randomized controlled trials of acupuncture and moxibustion combined with western medicine for the treatment of obsessive-compulsive disorder will be searched in the databases including PubMed, EMBASE, the Cochrane library, Web of science, China National Knowledge Infrastructure(CNKI), WanFang, the Chongqing VIP Chinese Science and Technology Periodical Database, and China biomedical literature database (CBM) from inception to June, 2020. In addition, Baidu, Google Scholar, International Clinical Trials Registry Platform, and Chinese Clinical Trials Registry will be searched to obtain the gray literature and relevant data that have not yet been published. Two qualified researchers will extract data and assess the risk of bias from included studies dependently. Statistical analysis is performed in RevMan 5.3 software.

**Results:**

The efficacy and safety of acupuncture and moxibustion combined with western medicine for obsessive-compulsive disorder will be assessed based on the total effective rate, Hamilton Anxiety Scale score, Hamilton Rating Scale for Depression score, Clinical Global Impression score, side effects, and so on.

**Conclusions:**

The proposed systematic review and meta-analysis of acupuncture and moxibustion combined with western medicine for treating obsessive-compulsive disorder is expected to provide reliable evidence for clinical application.

**Ethics and dissemination:**

The private information from individuals will not publish. This systematic review also will not involve endangering participant rights. Ethical approval is not required. The results may be published in a peer-reviewed journal or disseminated in relevant conferences.

**OSF Registration number:**

DOI 10.17605/OSF.IO/CDGTW

## 1 Introduction

Obsessive-compulsive disorder (OCD) is common, chronic mental disorder, which is characterized by recurrent, unwanted, or intrusive thoughts (obsessions) and repetitive behaviors or mental action (compulsions), affecting about 1% to 2% of the general population.^[1–3]^ The incidence of OCD in adolescents is higher in males than females, about 2:1. There is no significant sex difference in incidence rate among adults. However, OCD is common among mental workers.^[4]^ Epidemiological studies have shown that OCD can lead to severe damage to quality of life, mainly in terms of social functions.^[5]^ Some researches indicated that OCD was linked to serious impairment in various areas of quality of life including mental quality of life, social relations, family, or work life.^[6–12]^ Although the pathogenesis of OCD is unclear, it is likely to be the changes of corticostriato-thalamo-cortical circuitry including pivotal gray matter nodes.^[13,14]^ At present, OCD is mainly treated with western medicine, and the remission rate of drug treatment is only 40% to 60%. However, western medicine treatment has some shortcomings including side effects, long course of treatment, easy to relapse, and high cost.^[15]^ Therefore, it is urgent to explore an effective and safe alternative therapy for OCD.

Acupuncture and moxibustion, as a popular form of complementary and alternative therapy, have a good clinical efficacy all over the world.^[16]^ In traditional Chinese medicine, acupuncture and moxibustion are 2 inseparable therapies, the former is to stimulate acupoints with needles, the latter is to burn Artemisia Vulgaristhe and produce heat.^[17]^ Acupuncture and moxibustion can strengthen and tonify the brain. And they have the advantages of low side effects, high safety, and low cost.^[18]^

The research showed that acupuncture and moxibustion have a good clinical efficacy on OCD, with high safety and slight side effects, which may be related to the improvement of 5-hydroxytryptamine and dopamine (DA) functions in the brain of OCD patients.^[18,19]^ However, there is no systematic review assessing the efficacy and safety of acupuncture and moxibustion for OCD. Thus, the systematic review and meta-analysis is aimed to evaluate the efficacy and safety of acupuncture and moxibustion for OCD patients, providing reliable evidence for clinical application.

## 2 Methods

### 2.1 Study registration

This protocol of systematic review and meta-analysis has been drafted under the guidance of the preferred reporting items for systematic reviews and meta-analyses protocols (PRISMA-P). Moreover, it has been registered on open science framework (OSF) on June 22, 2020 (Registration number: DOI 10.17605/OSF.IO/CDGTW).

### 2.2 Ethics

Ethical approval is not required because there is no patient recruitment and personal information collection, and the data included in our study are derived from published literature.

### 2.3 Inclusion criteria for study selection

#### 2.3.1 Type of studies

Randomized controlled trials (RCTs) involving acupuncture and moxibustion for the treatment of OCD will be included. Blinding and publication status are not limited, but language will be restricted to Chinese and English.

#### 2.3.2 Type of participants

All participants diagnosed as OCD will be included regardless their country, age, race, and sex.

#### 2.3.3 Type of interventions

In the experimental group, OCD patients will be treated with acupuncture and moxibustion therapy (acupuncture, moxibustion, fire needle, auricular, electro-acupuncture, and so on) combined with western medicine. In the control group, OCD patients will be treated with western medicine alone.

#### 2.3.4 Type of outcome measures

The primary outcome is the total effective rate, as measured by Yale-Brown Obsessive Compulsive Scale (Y-BOCS scale).^[20]^ Partial disappearance of clinical symptoms and the reduction rate of Y-BOCS >25% are effective. If the clinical symptoms do not disappear or worsen, or the reduction rate of Y-BOCS is <25%, the efficacy evaluation is not effective. The total effective rate is the sum of apparent efficiency plus effective rate.

The secondary outcomes are Hamilton Anxiety Scale score, Hamilton Rating Scale for Depression score, Clinical Global Impression score, and side effects.

### 2.4 Exclusion criteria

1. If the study is published repeatedly, the one with the most complete data will be included.
2. Studies with missing data, and cannot get the data after contacting the author.
3. Studies with obviously wrong data.
4. Low-quality study will be excluded. According to the following criteria, the included study was evaluated as low-quality, moderate-quality and high-quality:^[21]^① if randomization or allocation concealment is evaluated as high risk of bias, regardless of other risks, it will be evaluated as low-quality study; ② when randomization and allocation concealment are evaluated as low risk of bias, and all other quality evaluation items are evaluated as low or unclear risk of bias, it will be rated as high-quality study; ③ if the study will not meet the standards of high quality and low quality, it is considered as moderate-quality research.

### 2.5 Search strategy

We will perform a comprehensive search in the databases including PubMed, EMBASE, the Cochrane library, Web of science, China National Knowledge Infrastructure (CNKI), WanFang, the Chongqing VIP Chinese Science and Technology Periodical Database (VIP), and China biomedical literature database (CBM) from inception to June, 2020. In addition, we will search in Baidu, Google Scholar, International Clinical Trials Registry Platform (ICTRP), and Chinese Clinical Trials Registry (ChiCTR) to obtain the gray literature and relevant data that have not yet been published. The search terms are as follows: “acupuncture,” “moxibustion,” “auricular,” and “obsessive-compulsive disorder,” and so on. The search strategy (PubMed) is shown in Table 1 Search strategy in PubMed database.

**Table 1:**
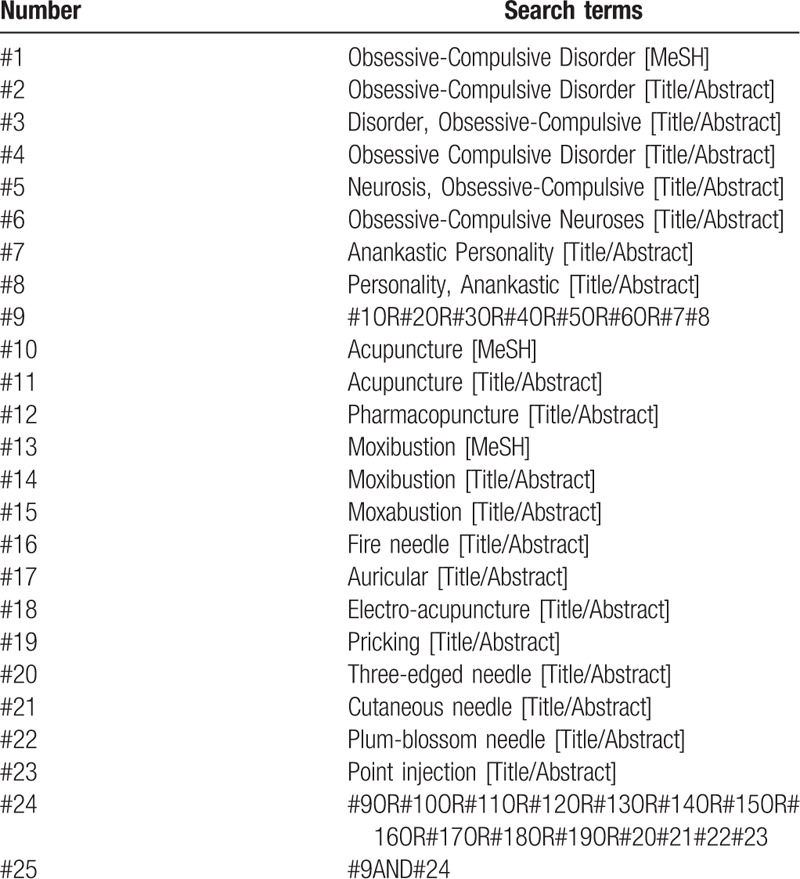
Search strategy in PubMed database.

### 2.6 Data extraction

**Figure 1:**
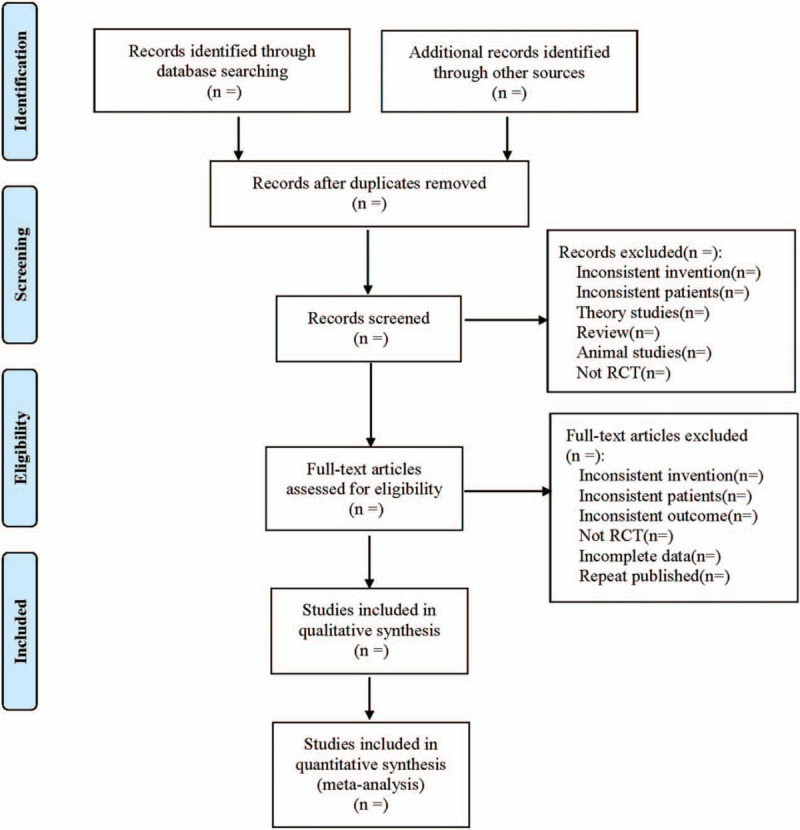
Flow diagram.

Searches were conducted in the above database according to guidance from The Cochrane Collaborative Network System Evaluator's Manual Version 5.0. Two researchers will select the literature by reading the title and abstract independently. Relevant studies will be initially included and imported into Endnote X7 for removal of duplications. Then, by reading the full text of the article, new screening is carried out by referring to the inclusion criteria and exclusion criteria. Two qualified researchers will extract data from included studies dependently. Any inconsistent views will be solved by a third researcher through discussion. The following information will be extracted: basic information of included studies (study title, first author, publication year, and so on); basic characteristics (sample size, age, sex ratio, and the course of disease between experimental group and control group); interventions (intervention measure, intervention time, frequency, drug dose, and pathway of included studies); the entry of risk of bias assessment; the outcomes and relevant measurement data. The literature screening process is shown (Fig. 1 Flow diagram).

### 2.7 Risk of bias assessment

The risk of bias for each eligible study will be assessed by 2 researchers dependently according to the Cochrane Collaboration's tool including 7 terms. According to these criteria (random sequence generation, allocation concealment, blinding, incomplete data, selective result reports and other bias), risk of bias is classified into the following levels: unclear, low, and high risk of bias. Any divergences will be solved through discussion by a third researcher.

### 2.8 Statistical analysis

#### 2.8.1 Data synthesis

Statistical analysis is performed in RevMan 5.3 software. For dichotomous outcome data, the risk ratio with 95% confidence intervals (CIs) is calculated. For continuous outcome data, if the measurement index unit or measurement tool is the same, the weighted mean difference (WMD) with 95% CIs is adopted; if the measurement index unit or measurement tool is different, the standard mean difference (SMD) with 95% CIs is used. Statistical heterogeneity will be checked by *P* value and *I*^*2*^ statistics. If *P* ≥.1, *I*^*2*^ ≤50%, there is no significant heterogeneity; thus, the fixed-effect model is used. When *P* < .1, *I*^*2*^≥50%, the sources of heterogeneity are analyzed, and subgroup analysis may be performed. If there is statistical heterogeneity between studies but no clinical heterogeneity, the random-effect model is selected to analyze. Otherwise, we exclude the study from meta-analysis.

#### 2.8.2 Dealing with missing data

When occurring missing, incomplete, and unclear data, we will contact the primary author to obtain it. If data are not acquired, we will analyze the present data and discuss its possible effect on the conclusion.

#### 2.8.3 Subgroup analysis

Subgroup analysis will be constructed based on the age of patients, the course of disease, and the intervention type including the type of acupuncture and moxibustion, the type of western medicine, and intervention time.

#### 2.8.4 Sensitivity analysis

Sensitivity analysis will be performed to measure the stability of study results by removing the study of high risk of bias.

#### 2.8.5 Reporting bias

When >10 studies are included, funnel plots and statistic tests are used to detect overall estimated reporting bias. Egger and Begg test are used to quantitatively assess potential publication bias.

#### 2.8.6 Evidence quality evaluation

The evidence evaluation of results is summarized by the Grading of Recommendations Assessment, Development and Evaluation (GRADE) system. The quality of evidence is divided into high, moderate, low, and very low. There are 8 factors that affect the quality of evidence. Among them, the factors that may reduce the quality of evidence are: risk of bias, inconsistency, indirectness, imprecision, publication bias; the factors that may improve the quality of evidence are: the large effect value, the confounding factors reducing the clinical efficacy, dose–response relationship. As we include RCTs, the default is a high-quality study. According to the above 8 factors, we will upgrade or downgrade.

## 3 Discussion

OCD belongs to the category of “Yu Zheng” (depression syndrome) in the traditional Chinese medicine. With the increasing pressure of life, the incidence rate has been increasing year by year. The first onset age of OCD is mostly around adolescence or adolescence.^[22]^ It is a kind of disease caused by the disorder of emotion and will, which leads to the obstruction of Qi, the loss of liver function and the disorder of viscera function, which seriously affects the patients’ mental health and quality of life.^[23]^

In recent years, many clinical explorations of acupuncture and moxibustion in the treatment of OCD have made some progress.^[18]^ Acupuncture and moxibustion treatment of OCD is mainly to invigorate the brain and open the mind. At present, there are mainly ordinary acupuncture, electroacupuncture, ear acupuncture, and so on.^[18]^ The common acupoints of acupuncture and moxibustion for the treatment of OCD are as follows: Baihui (DU20), Yintang (EX-HN3), Taiyang (EX-HN5), Neiguan (PC6), Sanyinjiao (SP6), Jiaji (EX-B2), and so on. It was found that acupuncture at Baihui (DU20) and Yintang (EX-HN3) points could effectively improve the behavior changes of rats with OCD, and its mechanism might be related to the decrease of 5-HT activity and DA content in the brain of rats with OCD.^[24]^ Some studies have shown that acupuncture or electroacupuncture is effective in the treatment of OCD, with faster effect and lower side effects than western medicine alone.^[22]^

However, so far, there is no systematic review and meta-analysis evaluating the efficacy and safety acupuncture and moxibustion for the treatment of OCD. Thus, it is important to evaluate the efficacy and safety of acupuncture and moxibustion for OCD patients, providing reliable evidence for clinical application. However, the study has some limitations. There are certain heterogeneity due to the type of acupuncture and moxibustion and difference of acupoints. And only studies published in English and Chinese are retrieved, so important studies published in other languages may be missed.

## Author contributions

Data collection: Jingyu Xu and Yang Huang.

Funding acquisition: Shenjun Wang and Ruiwen Song.

Resources: Wen Wang and Xinju Li.

Software: Chunying Tian.

Supervision: Shenjun Wang and Ruiwen Song.

Writing – original draft: Chunying Tian and Yihua Fan.

Writing – review & editing: Chunying Tian, Shenjun Wang,and Ruiwen Song.
